# Advances in Measurement Invariance and Mean Comparison of Latent Variables: Equivalence Testing and A Projection-Based Approach

**DOI:** 10.3389/fpsyg.2017.01823

**Published:** 2017-10-24

**Authors:** Ge Jiang, Yujiao Mai, Ke-Hai Yuan

**Affiliations:** Department of Psychology, University of Notre Dame, Notre Dame, IN, United States

**Keywords:** equivalence testing, measurement invariance, minimum tolerable size, projection method, scalar invariance

## Abstract

Measurement invariance (MI) entails that measurements in different groups are comparable, and is a logical prerequisite when studying difference or change across groups. MI is commonly evaluated using multi-group structural equation modeling through a sequence of chi-square and chi-square-difference tests. However, under the conventional null hypothesis testing (NHT) one can never be confident enough to claim MI even when all test statistics are not significant. Equivalence testing (ET) has been recently proposed to replace NHT for studying MI. ET informs researchers a size of possible misspecification and allows them to claim that measurements are practically equivalent across groups if the size of misspecification is smaller than a tolerable value. Another recent advancement in studying MI is a projection-based method under which testing the cross-group equality of means of latent traits does not require the intercepts equal across groups. The purpose of this article is to introduce the key ideas of the two advancements in MI and present a newly developed R package equaltestMI for researchers to easily apply the two methods. A real data example is provided to illustrate the use of the package. It is advocated that researchers should always consider using the two methods whenever MI needs to be examined.

## 1. Introduction

Reliable and valid measurements are key to social and behavioral sciences. When studying difference across groups, an equally important concept is measurement invariance (MI) or equivalence (Mellenbergh, 1989; Meredith, 1993; Millsap, 2011; Kim et al., 2012), which entails that measurements in different groups are comparable. Equivalent measurements are logical prerequisites to the evaluation of substantive hypotheses, regardless of whether the interest is as simple as a test of mean difference between groups or as complex as a test for possible changes of theoretical constructs across groups (Vandenberg and Lance, 2000). In particular, the observed or estimated cross-group difference can be simply due to different types of attributes being measured across populations, rather than the difference in the same attribute. Then, the observed cross-group difference is not interpretable nor valid for quantifying the cross-group difference on the target attribute.

The most widely used approach to examine MI is multi-group structural equation modeling (SEM) which relies on a sequence of chi-square and chi-square-difference tests (Sörbom, 1974; Horn et al., 1983; Meredith, 1993). With multi-group SEM, the test of MI typically starts with the equality of population covariance matrices across groups. Rejection of this equality does not imply that the groups are not comparable. A series of tests are then conducted to identify the source of non-equivalence (e.g., factor structure, factor loadings, etc.) and also to determine the degree of equivalence. Equality constraints are added in a logical order, and the models being tested also become increasingly more restrictive (Byrne, 2010). Two models are connected by each set of equality constraints: a base model and a nested (constrained) model. The normal-distribution-based maximum likelihood (NML) is typically used to estimate the models, and we also have a test statistic that approximately follows a chi-square distribution. The difference between the values of the test statistic at the base and restricted models is called the chi-square-difference statistic, which is commonly used to evaluate the plausibility of the constraints. The most widely used statistic is the likelihood ratio test statistic corresponding to NML estimation, which is also what we use in this article.

Two major concerns exist over the multi-group SEM approach to MI. First, there is a logical issue when the conventional null hypothesis testing (NHT) is used to establish equivalence of measurements. In every step of MI tests, whenever the chi-square or chi-square-difference statistic is not significant at a given level (e.g., α = 0.05), we move to the next step of the analysis by assuming that the current model under the null hypothesis holds. However, a non-significant test statistic does not imply that the involved model is correct or the involved components are invariant across groups. This is because NHT is constructed to reject the null hypothesis, and one can never be confident enough to claim equivalence even when all the statistics are not significant. Under such a practice, any violation against the previous hypotheses will be carried over to the next test. Yuan and Chan (2016) contains an example in which a sequence of tests for endorsing MI yields a rather different conclusion from that of testing the equality of covariance matrices across groups. Yuan and Bentler (2004) also showed that nested chi-square test is unable to control type I errors when the based model is misspecified, and the power of the test can also become rather weak.

Second, multi-group SEM approach for MI requires the intercepts of the manifest variables to be equal across groups before the means of latent constructs can be estimated (Sörbom, 1974). The cross-group equality of intercepts is commonly called *scalar invariance* (Horn and McArdle, 1992). The review by Vandenberg and Lance (2000) indicated that scalar invariance is rarely satisfied in practice. Marsh et al. (2017) also noted that “scalar invariance is an unachievable ideal that in practice can only be approximated.” However, without scalar invariance, the means of the latent constructs cannot be compared under the conventional setup. Such a requirement greatly limits the use of the multi-group SEM approach to mean comparison of latent variables.

To address the first issue regarding the use of NHT, Yuan and Chan (2016) recently proposed using equivalence testing (ET) to replace NHT in multi-group SEM. In a sequence of tests for MI under ET, researchers are informed about a possible misspecification in every step, which enables them to effectively control the size of misspecification. Researchers can evaluate their results based on their own degrees of tolerance or using the adjusted cutoff values in connection with established rules of labeling the goodness of model fit in SEM. Yuan and Chan (2016) illustrated their approach using a simulated example with 2 groups, 9 variables, and 3 latent factors. They also provided an R program to compute the minimum tolerable size and adjusted cutoff values of fit indices for evaluating the goodness of the model under ET. However, one has to use a separate program to estimate the SEM model under different constraints before conducting ET using their R program. Thus, it is rather difficult for substantive researchers to correctly perform or interpret results at each step of the sequence of conducting the tests for MI. Also, although ET has been used in many areas of psychological and educational research, there is no self-contained software for conducting ET using chi-square and chi-square difference tests, especially for the purpose of MI. Our experience indicates that a statistical package must be in place before any new cutting-edge methodology can be applied by substantive researchers. Thus, we have developed an all-in-one R package equaltestMI that will be introduced in this article. Our illustration of the package with real data will also contribute to promoting ET in substantive areas where MI is routinely used in group comparison.

To address the second issue with the multi-group SEM approach to MI, Deng and Yuan (2016) proposed a new projection method to circumvent the scalar-invariance assumption by decomposing the observed means of the manifest variables into two orthogonal components. One component represents the means of the common scores and the other represents the mean of the specific factors. These two components are uniquely identified although the means of specific factors have been ignored in conventional factor analysis (Harman, 1976; Gorsuch, 1983). As we will see, the projection method allows us to test the cross-group equality of latent means independently from that of specific factors, and there is no need to constrain intercepts to be equal in this approach. In particular, only factor loadings are required equal across groups for conducting mean comparison of latent constructs. However, Deng and Yuan (2016) only presented the projection method using conventional NHT, not ET. Thus, the method still has the logical issues inherited from NHT, which will be addressed in this article by putting the projection method under ET.

The contributions of the current article are as follows: (1) using plain language to introduce the key ideas of ET and the projection method for examining MI; (2) combine ET and the projection method to provide valid inference on the tests of equality of latent factors and specific factors; (3) developing an accompanying R package equaltestMI so that substantive researchers can easily apply the two new methods as well as combining them in conducting MI analysis; and (4) providing a detailed tutorial to illustrate the use of equaltestMI with a real data example.

In the following sections, we first briefly review the types of tests in the conventional approach to MI. Then, we introduce the ET framework and the projection method. Next, we provide a step-by-step tutorial to illustrate the use of the accompanying R package equaltestMI with a real data example. We conclude this article with some remarks on the two new methods and the use of the R package.

## 2. Methods

This section introduces two recent methodological advancements in examining MI. By avoiding the logical problem and unrealistic assumptions with the conventional multi-group SEM approach, the new methods provide a more valid platform for studying MI. In particular, ET is proposed to replace the NHT framework and the projection method is proposed to replace the tests of mean structure under the conventional multi-group SEM as developed in Sörbom (1974). To help introduce the two new methods, we first review the models and notations used in multi-group SEM and the sequence of tests for examining MI.

### 2.1. Multi-group SEM

Suppose a set of *p* variables are collected for each of *m* groups, and they are obtained by administering the same instrument or properly adjusted to be on the same scale. Let **x**^(*j*)^ represent the vector of variables in the population for group *j*, *j* = 1, .., *m*, and the following SEM model holds within each group:

(1) $$\begin{array}{l} {\text{x}}^{\left(j\right)}=\gamma^{\left(j\right)}+\Lambda^{\left(j\right)}\xi^{\left(j\right)}+\varepsilon^{\left(j\right)},j=1,\cdots \;,m, \end{array}$$

where the superscript (*j*) indicates the group membership; **γ**^(*j*)^ is a vector of *p* intercepts of the manifest variables, Λ^(*j*)^ is *p* × *k* matrix of factor loadings, **ξ**^(*j*)^ is a vector of *k* factor scores, and **ε**^(*j*)^ is a vector of *p* errors. We assume that errors are uncorrelated and Ψ^(*j*)^ = Cov(**ε**^(*j*)^) is a diagonal matrix. The errors and the factors are also assumed to be uncorrelated with *E*(**ξ**^(*j*)^) = **τ**^(*j*)^ and Cov(**ξ**^(*j*)^) = Φ^(*j*)^. It follows from Equation (1) that the model-implied mean and covariance structures for the *m* groups are respectively

(2) $$\begin{array}{l} \mu^{\left(j\right)}=\gamma^{\left(j\right)}+\Lambda^{\left(j\right)}\tau^{\left(j\right)}\text{ and}\\ \Sigma^{\left(j\right)}=\Lambda^{\left(j\right)}\Phi^{\left(j\right)}\Lambda \prime^{\left(j\right)}+\Psi^{\left(j\right)},j=1,\cdots ,m. \end{array}$$

Note that different groups might have different structures in (2), and **γ**^(*j*)^, Λ^(*j*)^, **τ**^(*j*)^, Φ^(*j*)^, and Ψ^(*j*)^ are free to vary. The key point here is that the latent variables ξ^(*j*)^ cannot be directly observed and must be measured with a set of manifest variables. These are standard assumptions in structural equation modeling and factor analysis, not particular to MI. With these notations, the steps of tests of MI (Vandenberg and Lance, 2000) and their corresponding chi-square and chi-square-difference statistics are given in Table 1. In the table, each subscript of the letter *H* represents the hypothesis for the involved parameters; and the subscripts of *T* represent the joint hypotheses under which the statistic is computed; while the superscript of *T* represents the hypothesis being tested by the nested chi-square statistic.

**Table 1:** Types and steps of tests with the conventional approach to measurement invariance.

**Step**	**Hypothesis**	**Name**	**Test statistics**	
			**Overall model**	**Nested model**
1	$H_{\sigma }:\Sigma^{\left(1\right)}=\cdots =\Sigma^{\left(m\right)}$		*T*_σ_	
2	$H_{c}:\Sigma^{\left(j\right)}=\Sigma \left(\theta^{\left(j\right)}\right)$	configural	*T*_*c*_	
3	$H_{\lambda }:\Lambda^{\left(1\right)}=\cdots =\Lambda^{\left(m\right)}$	metric	*T*_*c*λ_	$T_{c}^{\lambda }=T_{c\lambda }-T_{c}$
4a	$H_{\psi }:\Psi^{\left(1\right)}=\cdots =\Psi^{\left(m\right)}$		*T*_*c*λψ_	$T_{c\lambda }^{\psi }=T_{c\lambda \psi }-T_{c\lambda }$
5a	$H_{\phi }:\Phi^{\left(1\right)}=\cdots =\Phi^{\left(m\right)}$		*T*_*c*λψϕ_	$T_{c\lambda \psi }^{\phi }=T_{c\lambda \psi \phi }-T_{c\lambda \psi }$
4b	$H_{\gamma }:\gamma^{\left(1\right)}=\cdots =\gamma^{\left(m\right)}$	scalar	*T*_*c*λγ_	$T_{c\lambda }^{\gamma }=T_{c\lambda \gamma }-T_{c\lambda }$
5b	$H_{\tau }:\tau^{\left(1\right)}=\cdots =\tau^{\left(m\right)}$		*T*_*c*λγτ_	$T_{c\lambda \gamma }^{\tau }=T_{c\lambda \gamma \tau }-T_{c\lambda \gamma }$
4c	$H_{\gamma }:\gamma^{\left(1\right)}=\cdots =\gamma^{\left(m\right)}$	scalar	*T*_*c*λγ_	$T_{c\lambda }^{\gamma }=T_{c\lambda \gamma }-T_{c\lambda }$
5c	$H_{\psi }:\Psi^{\left(1\right)}=\cdots =\Psi^{\left(m\right)}$		*T*_*c*λγψ_	$T_{c\lambda \gamma }^{\psi }=T_{c\lambda \gamma \psi }-T_{c\lambda \gamma }$
6c	$H_{\tau }:\tau^{\left(1\right)}=\cdots =\tau^{\left(m\right)}$		*T*_*c*λγψτ_	$T_{c\lambda \gamma \psi }^{\tau }=T_{c\lambda \gamma \psi \tau }-T_{c\lambda \gamma \psi }$

Following the work of Sörbom (1974) and Jöreskog (1971), the tests of MI usually start with a test of equality of the population covariance matrices. Statistically speaking, the first step tests $H_{\sigma }:\Sigma^{\left(1\right)}=\cdots =\Sigma^{\left(m\right)}$, where Σ^(*j*)^ is the population covariance matrix of group *j*. A non-significant statistic of this test is generally regarded as an endorsement of overall measurement equivalence. However, a significant test statistic does not mean that the involved groups are not comparable and it is necessary to conduct subsequent tests to identify the sources of non-equivalence. To test if any aspects of the groups are invariant, a common SEM model is assumed and the equalities of its components across groups are tested in an increasingly restrictive fashion. In step 2, the SEM model is fitted to each group separately and one examines if the same model structure holds across groups (configural invariance). We denote configural invariance as $H_{c}:\Sigma^{\left(j\right)}=\Sigma \left(\theta^{\left(j\right)}\right)$, *j* = 1, ⋯ , *m*, implying that the same structured model Σ(**θ**^(*j*)^) holds in all the groups but their parameters **θ**^(*j*)^ can differ across groups. If *H*_*c*_ holds, configural invariance is established, and one tests the equality of factor loading matrices (metric invariance) in step 3. We denote metric invariance as $H_{\lambda }:\Lambda^{\left(1\right)}=\cdots =\Lambda^{\left(m\right)}$, implying that the factor loadings are invariant across all the groups. After both configural (*H*_*c*_) and metric invariances (*H*_λ_) are established, one next separately tests the equalities in covariance structure and mean structure. For the covariance structure, one first tests the equality of error variances Ψ^(*j*)^ across groups; and if that holds, one then tests the equality of factor covariance matrices Φ^(*j*)^ across groups.

For the mean structure, two types of invariance have been conceptualized (Meredith, 1993; Vandenberg and Lance, 2000). Measurements satisfying Steps 2, 3, and 4b are called *strong invariance* (Meredith, 1993), while those satisfying Steps 2, 3, 4c, and 5c are called *strict invariance*. For either of the invariances, the equality of intercepts of manifest variables (scalar invariance, *H*_γ_) is tested first. If scalar invariance holds, strong invariance is achieved, and one continues to test the equality of latent means (*H*_τ_). To achieve strict invariance, one needs to test the equality of error variances Ψ^(*j*)^ after scalar invariance and then tests the equality of latent means. In summary, the steps to examine MI of covariance structure is 1 → 2 → 3 → 4*a* → 5*a*, the sequence for testing mean structure and achieving strong invariance is 1 → 2 → 3 → 4*b*, and the sequence for achieving strict invariance is 1 → 2 → 3 → 4*c* → 5*c*. Step 5b or 6c might not be needed if the interest of the MI analysis is to compare individuals. But the test of *H*_τ_ will be the ultimate goal if the interest is to compare groups, as in ANOVA or *t*-test.

To test the hypotheses mentioned above, chi-square and chi-square-difference statistics are computed in the last two columns of Table 1 with the superscripts and subscripts denoting the involved hypotheses. In any of the three sequences above, a model with more constraints is nested in a model with fewer constraints and the additional constraints can be tested using the chi-square-difference statistic. For example, the statistic *T*_*c*λγ_ in step 4b of Table 1 evaluates the joint hypothesis *H*_*c*λγ_ = *H*_*c*_ + *H*_λ_ + *H*_γ_, and the model under *H*_*c*λγ_ is nested in the model under *H*_*c*λ_. The corresponding chi-square-difference statistic $T_{c\lambda }^{\gamma }$ evaluates the additional constraints under *H*_γ_, and it is computed as the difference between *T*_*c*λγ_ and *T*_*c*λ_, i.e., $T_{c\lambda }^{\gamma }=T_{c\lambda \gamma }-T_{c\lambda }$.

### 2.2. Equivalence testing

ET was proposed to address the logical issues with NHT to establish equivalence of measures across groups (Yuan and Chan, 2016). A major distinction between ET and NHT is the formulation of null hypothesis. The null hypothesis under NHT is that the model or constraints hold in the population, whereas the null hypothesis under ET is that the size of misspecification in the model or constraints is greater than a tolerable value. When the null hypothesis is rejected under ET at level α, we are confident with probability 1 − α that the size of misspecification is less than or equal to the tolerable value. Consequently, the current model or components are deemed^1^ as MI, and we continue with testing the subsequent hypothesis. Otherwise, we declare that the size of misspecification in the current model or hypothesis is not tolerable and stop at the previous level of equivalence. We will further discuss the specification of tolerable values using the fit index RMSEA (root mean square error of approximation, Steiger and Lind, 1980).

As with NHT, we need to have a statistic to work with under ET. In this article, we use the likelihood ratio statistic *T*_*ml*_ = (*N* − *m*)*F*_*ml*_, where *N* is the total sample size across the *m* groups and *F*_*ml*_ is the normal-distribution-based discrepancy function proportionally weighted according to the sample sizes in the *m* groups (e.g., Equations 23 and 4 in Yuan and Bentler, 2006). Let *F*_*ml*0_ be the population counterpart of *F*_*ml*_, the null hypothesis under NHT is *H*_0_:*F*_*ml*0_ = 0 whereas that under ET is

(3) $$\begin{array}{l} H_{e0}:F_{ml0}>\epsilon_{0} \end{array}$$

with ϵ_0_ being a small positive number that one can tolerate for the size of misspecification. As for NHT, we need to assume that *T*_*ml*_ follows a central chi-square distribution $\chi_{df}^{2}$ when *F*_*ml*0_ = 0 and a non-central chi-square distribution $\chi_{df}^{2}\left(\delta \right)$ when *F*_*ml*0_ > 0, where δ = (*N* − *m*)*F*_*ml*0_ is the non-centrality parameter (ncp). Let δ_0_ = (*N* − *m*)ϵ_0_ and *c*_α_(δ_0_) be the left-tail critical value of $\chi_{df}^{2}\left(\delta_{0}\right)$ at level α. Then we reject the null hypothesis *H*_*e*0_ in (3) when *T*_*ml*_ < *c*_α_(δ_0_) and the type I error is controlled at level α. When the *H*_*e*0_ in (3) is rejected, we conclude that the size of misspecification of the current model is no greater than ϵ_0_ with 1 − α confidence.

Similarly, when the chi-square-difference statistic is formulated according to *T*_*ml*_, and our null hypothesis under ET is

(4) $$\begin{array}{l} H_{eab}:F_{mla0}-F_{mlb0}>\epsilon_{0ab}, \end{array}$$

where ϵ_0*ab*_ is a tolerable value of misspecification due to the additional constraints in model *A* beyond that in the based model *B*. When the difference statistic is smaller than the left-tail critical value corresponding to $\chi_{dfab}^{2}\left(\delta_{0ab}\right)$ with δ_0*ab*_ = (*N* − *m*)ϵ_0*ab*_, we reject *H*_*eab*_ and conclude with probability 1 − α that the size of misspecification due to the additional constraints in model *A* (beyond that in model *B*) is smaller than the tolerable value or is tolerable.

The specification of a tolerable value ϵ_0_ is crucial for ET. Although any choice of ϵ_0_ cannot avoid an arbitrary nature, it is a necessary element for conducting ET. Following Yuan and Chan (2016), we specify ϵ_0_ by relating it to the population value of the fit index RMSEA through

(5) $$\begin{array}{l} \epsilon_{0}=df{\left({\text{RMSEA}}_{0}\right)}^{2}/m, \end{array}$$

where ${\text{RMSEA}}_{0}={\left\{m\delta_{0}/\left[df\left(N-m\right)\right]\right\}}^{1/2}$ with δ_0_ being the ncp of the nominal chi-square distribution corresponding to the statistic *T*_*ml*_ with *m* groups (Steiger, 1998). With respect to the use of the conventional^2^ RMSEA, MacCallum et al. (1996) suggested cutoff^3^ values 0.01, 0.05, 0.008, and 0.10 to distinguish between excellent, close, fair, mediocre, and poor fit, respectively. As can be seen from Equation (5), when other terms are held constant, the larger the value of ϵ_0_, the larger the RMSEA_0_ is. This means that for a given model, a larger tolerable value of misspecification ϵ_0_ implies that we allow for a less ideal model as quantified by RMSEA_0_. There are two ways to use the relationship in Equation (5) to evaluate the fit of the current model. One can obtain the values of ϵ_0_ corresponding to RMSEA_0_ = 0.01, 0.05, 0.08, and 0.10, respectively, and compare *T*_*ml*_ against the critical values *c*_α_(ϵ_0_) with those ϵ_0_ values. If *T*_*ml*_ is between the critical values corresponding to RMSEA_0_ = 0.01 and 0.05, then the model achieves a close fit for the observed samples.

Alternatively, we can solve the equation

(6) $$\begin{array}{l} T_{ml}=c_{\alpha }\left(\epsilon_{t}\right). \end{array}$$

for the value of ϵ_*t*_, which is an increasing function of *T*_*ml*_. Unlike the ϵ_0_ in Equation (3) or (5) that is specified a priori, the ϵ_*t*_ in (6) is data dependent. However, rejection of the hypothesis in (3) is equivalent to ϵ_*t*_ < ϵ_0_. Yuan and Chan (2016) called the ϵ_*t*_ in (6) *the minimum tolerable size* (T-size) of misspecification. If one cannot tolerate the T-size ϵ_*t*_, then hypothesis with any prespecified ϵ_0_ that is less than ϵ_*t*_ cannot be rejected since *T*_*ml*_ > *c*_α_(ϵ_0_), and we will not be able to continue with the analysis in the sequence of endorsing MI. Let RMSEA_*t*_ be the value of RMSEA defined at ϵ_*t*_. ET can be equivalently conducted using the established cutoff values of RMSEA and the values of RMSEA_*t*_ corresponding to the ϵ_*t*_ in (6). We will illustrate this procedure in a later section via a real data example.

Compared with the conventional methods, ET informs us the size of a possible misspecification at each step of endorsing MI, and it is still up to the researcher to decide whether the size is tolerable. Established values of RMSEA facilitate us to make a decision on the size of misspecification according to the values of RMSEA_*t*_. However, the conventional cutoff values of RMSEA are too stringent to evaluate the model fit under ET, and the cutoff values need to be modified accordingly. Yuan and Chan (2016) developed formulas of adjusted cutoff values for evaluating RMSEA_*t*_ so that labeling of goodness of fit is comparable to evaluating the conventional RMSEA by existing cutoff values. These formulas are incorporated in our R package and new cutoffs will be used in the real data example. Technical details and formulas can be found in Yuan and Chan (2016).

### 2.3. Projection method

One major goal of MI is to test the cross-group equality of means of latent traits, especially when our interest is to study the effect of different experimental conditions or group difference. However, with the conventional approach, the test of *H*_τ_ in Table 1 or even the estimation of **τ** requires the hypothesis *H*_γ_ to hold, which is theoretically unnecessary and practically hard to achieve. In this subsection, we introduce a new setup proposed in Deng and Yuan (2016) under which the means of the latent traits can be compared even when the intercepts of manifest variables are not equal across groups. A projection method is used so that the means of manifest variables in each group are decomposed into orthogonal components of common scores and specific factors. The test of cross-group equality of the means of the common scores is essentially the test of cross-group equality of means of latent traits under the conventional setup whereas the test of cross-group equality of means of specific factors is related to but different from the test of cross-group equality of the intercepts under the conventional setup.

In the conventional setup of examining MI via Equation (1), the mean structure involves the intercepts and the means of the latent traits. The intercepts **γ**^(*j*)^ need to be set as equal across groups so that the means **τ**^(*j*)^ = *E*(**ξ**^(*j*)^) can be identified and estimated (Sörbom, 1974). Similarly, the means **τ**^(*j*)^ of one group need to be set at **0** as the baseline so that the **τ**^(*j*)^ of the other groups are the differences from those of the baseline group. To circumvent this assumption, Deng and Yuan (2016) proposed to decompose the observed variables into common scores, specific factors, and measurement errors

(7) $$\begin{array}{l} {\text{x}}^{\left(j\right)}=\Lambda {\text{f}}^{\left(j\right)}+{\text{u}}^{\left(j\right)}+{\text{e}}^{\left(j\right)},j=1,\cdots \;,m, \end{array}$$

where Λ**f**^(*j*)^ represents the vector of *p* common scores, **u**^(*j*)^ represents the vector of *p* specific factors, and **e**^(*j*)^ is a vector of *p* measurement errors, with *E*[**f**^(*j*)^] = **κ**^(*j*)^, *E*[**u**^(*j*)^] = **ν**^(*j*)^, and *E*[**e**^(*j*)^] = **0**. There is no superscript on the factor loading matrix Λ because the decomposition in Equation (7) is a step following metric invariance $H_{\lambda }:\Lambda^{\left(1\right)}=\cdots =\Lambda^{\left(m\right)}=\Lambda$. When metric invariance does not hold, researchers have the option to identify a subset of variables that satisfy metric invariance (Byrne et al., 1989; Millsap and Kwok, 2004). Then the projection method can be equally applied to the identified subset, as was discussed in Deng and Yuan (2016).

Note that the new setup in Equation (7) is not a simple reparameterization of the conventional setup in Equation (1). In fact, the interpretation has changed entirely. With the projection method, we assume that the space of common score is orthogonal to that of specific factors, and the comparison of means of the common scores or factors **f**^(*j*)^ is conducted independently from those of **u**^(*j*)^. Under the new setup, the mean structure of **x**^(*j*)^ is decomposed as

(8) $$\begin{array}{l} \mu^{\left(j\right)}=\mu_{\kappa }^{\left(j\right)}+\nu^{\left(j\right)}, \end{array}$$

where $\mu_{\kappa }^{\left(j\right)}=\Lambda \kappa^{\left(j\right)}$ is the part of **μ**^(*j*)^ = *E*(**x**^(*j*)^) that is projected onto the space of common scores, and **ν**^(*j*)^ is the part of **μ**^(*j*)^ that is projected onto the space of specific factors. The two components are identified once Λ is identified. Regardless of the values of **κ**^(*j*)^, $\mu_{\kappa }^{\left(j\right)}$ is always the linear combinations of the columns of Λ.

Let $\hat{\Lambda }$ be the estimated factor loading matrix and ${\bar{\text{x}}}^{\left(j\right)}$ be the sample means of the *j*th group. Then the space of the estimated common scores consists of vectors of linear combinations of the columns of $\hat{\Lambda }$, and is totally determined by $\hat{\Lambda }$. The estimated means of the common scores are consequently obtained by projecting ${\bar{\text{x}}}^{\left(j\right)}$ onto the column space of $\hat{\Lambda }$, and we denote it as ${\hat{\mu }}_{\kappa }^{\left(j\right)}$. Similarly, the estimated means of the specific factors are obtained by projecting ${\bar{\text{x}}}^{\left(j\right)}$ onto the space that is orthogonal to that of $\hat{\Lambda }$, and we denote it as ${\hat{\nu }}^{\left(j\right)}$. Details of the projection matrix and examples are provided in Deng and Yuan (2016). In particular, there exists ${\bar{\text{x}}}^{\left(j\right)}={\hat{\mu }}_{\kappa }^{\left(j\right)}+{\hat{\nu }}^{\left(j\right)}$. Also, an estimate of **κ**^(*j*)^ is uniquely obtained from ${\hat{\mu }}_{\kappa }^{\left(j\right)}$, and we denote it as ${\hat{\kappa }}^{\left(j\right)}$. Thus, the estimates of means of common and specific factors only depend on the sample means and estimated common factor loading matrix, and do not involve estimating the intercepts in Equation (1).

Two types of invariance tests on means can be conducted under the new setup. One test is about cross-group equality of means of common scores, which is equivalent to the test on cross-group equality of means of the latent constructs. The other test is on cross-group equality of means of specific factors. The corresponding hypotheses are

(9) $$\begin{array}{l} H_{\kappa }:\kappa^{\left(1\right)}=\cdots =\kappa^{\left(m\right)}\text{or}\mu_{\kappa }^{\left(1\right)}=\cdots =\mu_{\kappa }^{\left(m\right)}, \end{array}$$

and

(10) $$\begin{array}{l} H_{\nu }:\nu^{\left(1\right)}=\cdots =\nu^{\left(m\right)}. \end{array}$$

The two hypotheses can also be formulated as $H_{\kappa }:\kappa_{d}^{\left(j\right)}=\kappa^{\left(j\right)}-\kappa^{\left(1\right)}=0$ and $H_{\nu }:\nu_{d}^{\left(j\right)}=\nu^{\left(j\right)}-\nu^{\left(1\right)}=0$, *j* = 2, ⋯ , *m*. Deng and Yuan (2016) showed that ${\hat{\kappa }}_{d}^{\left(j\right)}$ and ${\hat{\nu }}_{d}^{\left(j\right)}$ asymptotically follow normal distributions, and each of the two hypotheses can be tested using a Wald^4^ statistic *T*_*gls*_ = *NF*_*gls*_ that asymptotically follows a chi-square distribution with degrees of freedom *df*_κ_ = (*m* − 1)*k* and *df*_ν_ = (*m* − 1)(*p* − *k*), respectively. In addition to using the Wald statistics, the two hypotheses in (9) and (10) can also be tested via the bootstrap methodology, especially when the sample sizes are not large enough.

The interest of mean comparison in most studies might be to find a significant difference across groups. If this is the goal, then conventional NHT would be logically sufficient and ET is not needed. However, it is hard to imagine that the population means of different groups are literally identical. A non-significant result might be due to a small sample size and/or a small effect size. Knowing the size of the difference would be more informative even if one cares primarily about significant differences. The framework of ET would not only inform researchers the size of a possible misspecification but also provide a confidence level to it. For ET, the two hypotheses in (9) and (10) need to be reformulated, parallel to Equation (3). That is, the null hypothesis for endorsing the equality of the **κ**^(*j*)^, *j* = 1, 2, ⋯ , *m*, becomes

(11) $$\begin{array}{l} H_{e\kappa }:F_{gls0}>\epsilon_{0}, \end{array}$$

where *F*_*gls*0_ is the population value of *F*_*gls*_ corresponding to the *T*_*gls*_ for testing *H*_κ_. Then the critical value for judging the significance of *T*_*gls*_ is the left-tail quantile of $\chi_{df_{\kappa }}^{2}\left(\delta_{0}\right)$ corresponding to level α, where δ_0_ = *Nϵ*_0_. We reject *H*_*eκ*_ when *T*_*gls*_ is smaller than the critical value. Similarly, we can test *H*_*eν*_ under ET, although there might be less interest in comparing the means of specific factors. As with the chi-square-difference statistics in the previous subsection, we can specify the value of ϵ_0_ via ${\text{RMSEA}}_{0}={\left(\epsilon_{0}/df_{\kappa }\right)}^{1/2}$ as well as by testing *H*_*eκ*_ using the T-size RMSEA^5^ corresponding to the Wald statistic. In our package equaltestMI, we compute the T-size RMSEA_*t*_ corresponding to the value of the Wald statistic instead of reporting the critical value $\chi_{df_{\kappa }}^{2}\left(\delta_{0}\right)$. Researchers can compare the value of RMSEA_*t*_ against the adjusted cutoff values, which are printed out in the output of the R package.

A key feature of the projection method is a validity index. Let $\mu_{d\kappa }^{\left(j\right)}=\Lambda \left(\kappa^{\left(j\right)}-\kappa^{\left(1\right)}\right)$, *j* = 2, ⋯ , *m*, and $\mu_{\kappa }^{\left(d\right)}$ is the vector of length *p*(*m* − 1) formulated by stacking the $\mu_{d\kappa }^{\left(j\right)}$; and **ν**^(*d*)^ is the vector of length *p*(*m* − 1) formulated by stacking the $\nu_{d}^{\left(j\right)}$, *j* = 2, 3, ⋯ , *m*. Deng and Yuan (2016) defined a validity index for mean difference as

(12) $$\begin{array}{l} \rho_{c}^{2}=\frac{|\mu_{\kappa }^{\left(d\right)}{|}^{2}}{|\mu_{\kappa }^{\left(d\right)}{|}^{2}+|\nu^{\left(d\right)}{|}^{2}}, \end{array}$$

where $|\mu_{\kappa }^{\left(d\right)}{|}^{2}$ and |**ν**^(*d*)^|^2^ denote the sums of squares of the elements in $\mu_{\kappa }^{\left(d\right)}$ and **ν**^(*d*)^, respectively. This validity index gives the percentage of the mean differences of the manifest variables that is due to the differences in means of the common scores. If the sample estimate ${\hat{\rho }}_{c}^{2}$ is not large enough, say less than 0.5, then items in the test might need to be modified or the administration of the data collection process might not be conducted properly. We will call $\rho_{c}^{2}$ the validity index for mean differences, because elaboration on the observed mean differences might be off the target when ${\hat{\rho }}_{c}^{2}$ is not sufficiently large, say greater than 0.70. In particular, when most of the mean differences in the manifest variables are not due to those in the latent traits, the validity of the measurements might be questionable. Then the empirical meaning of the observed differences will be different from the truth, which will create interpretational confounding. The extent to which the observed mean differences reflect the mean differences of the latent variables is not available following the analysis of the mean structures in the conventional setup, where cross-group equality of intercepts is a prerequisite for estimating mean differences of latent variables.

## 3. Real data example

In this section, we introduce the R package equaltestMI and illustrate its use via a real data example. Both ET and the projection method are implemented in the R package, which is available on CRAN and can be used on any R platform with version 3.1.0 or above. The development of equaltestMI relies on R packages lavaan (Rosseel, 2012) for obtaining chi-square statistics of invariance tests and semTools (semTools Contributors, 2016) for computing chi-square-difference tests and fit indices. The function for computing adjusted RMSEA cutoff values for ET is adapted from the R codes available at http://www3.nd.edu/~kyuan/mgroup/Equivalence-testing.R. The input to equaltestMI can be either raw data sets with group membership indicator or sample means and covariances.

### 3.1. Data set

**Figure 1:**
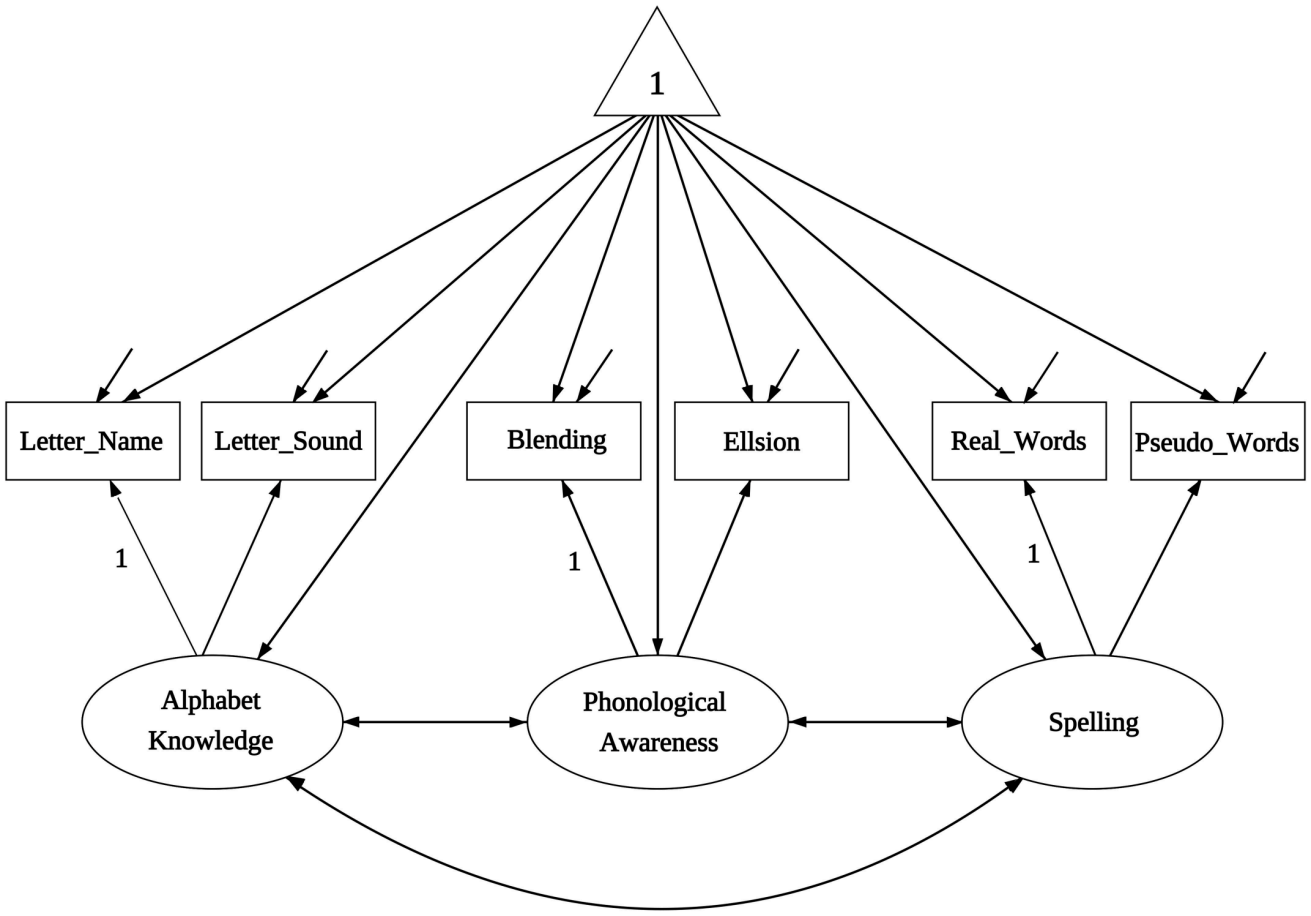
The path diagram for the model of Lee and Al Otaiba (2015).

Literacy-related difficulties for many children are due to lack of exposure to print or instructional resources, and thus socioeconomic status (SES) is an important demographic variable that strongly relates to academic achievement. The data we use for the illustration are from Lee and Al Otaiba (2015), and their Table 1 contains sample statistics (sample sizes, means, covariances) on early literacy skills from 2 sociodemographic groups of kindergartners, with *N*_1_ = 78 boys ineligible for free or reduced-price lunch (FRL) and *N*_2_ = 174 boys eligible for FRL. The interest of Lee and Al Otaiba is whether measurements on literature proficiency are invariant when compared students with lower SES (eligible for FRL) against those with higher SES (ineligible for FRL). There are six manifest variables in measuring literacy constructs: (1) letter-name fluency, (2) letter-sound fluency, (3) blending, (4) elision, (5) real words spelling, and (6) pseudo-words spelling. Following from Snow's (2006) definition of componential skills and the work of Schatschneider et al. (2004) on National Early Literacy Panel (NELP), the six variables aim to measure three aspects of literacy constructs: (1) alphabet knowledge, which refers to children's familiarity with letter forms, names, and corresponding sounds; (2) phonological awareness, which encompasses the ability to detect, manipulate, or analyze sounds in spoken language in varying complexities such as words, syllables, and phonemes; and (3) spelling, which measures the ability to spell words with letters (Piasta and Wagner, 2010). As indicated in Figure 1, alphabet knowledge, phonological awareness, and spelling are the three latent constructs behind the six variables. Lee and Al Otaiba (2015) examined the MI issues using the conventional methods. Let the boys who are ineligible for FRL be group one and those eligible for FRL be group two. We will use the six-variable-two-group model to illustrate the application of the new methods via the package equaltestMI.

### 3.2. Package equaltestMI

To use equaltestMI for the first time, one needs to download the package from CRAN and load it into R environment. This can be done by entering the following commands in R:

**Table:** 

install.packages(equaltestMI)	1
library(equaltestMI)	2

The line numbers in the right margin are for convenience of explaining the codes in our illustration, not part of the R commands. After loading the package equaltestMI into R, there is no need to load lavaan and semTools separately since they are listed as dependent packages of equaltestMI. However, one does need to have the two packages installed before the library command on line 2, otherwise equaltestMI cannot be successfully loaded.

The package equaltestMI has multiple R functions. The one that is routinely used is eqMI.main(). Other functions can be used to test the cross-group equality of population covariance matrices or to obtain adjusted RMSEA cutoff values in a separate analysis. Interested users are referred to supplementary material (http://www3.nd.edu/~kyuan/eqMI/Supplementary_Material_MI.pdf) and the page of equaltestMI on CRAN (https://cran.r-project.org/web/packages/equaltestMI/index.html), where details for using different functions are documented.

Different arguments can be provided to eqMI.main() for customized analysis. However, data input (raw data or sample means and covariances) has to be in required format. For a raw data set, column represents variables and row represents observations, and an additional column of duplicated numbers for group membership is needed for all the involved samples. For either raw data or sample statistics, the first row needs to be variable names, and including a group-membership indicator. If input data are sample statistics, the sample means must be stored in the format of vectors immediately following the variables names; and sample covariance matrix stored in the format of matrix are next, see **Appendix A** for the format. In particular, the first row of each file must have a space before the first variable name. The label 'mean' must be included prior to the numerical values of the mean vector. In addition, variable names are also needed in the first column of the covariance matrix as required by lavaan, these will be further used to in the model syntax, to be presented below.

For conducting the tests of MI, we first need to import the sample means and sample covariance matrices into R. This is done by the following R codes for this example:

**Table:** 

setwd(“C:/research/equaltestMI”)	3
Groupl <- read.table('Group1.txt', header = TRUE)	4
Group2 <- read.table('Group2.txt', header = TRUE)	5
Group1 <- as.matrix(Group1)	6
Group2 <- as.matrix(Group2)	7
M1 <- Group1[1,]	8
M2 <- Group2[1,]	9
Cov1 <- Group1[2:7,]	10
Cov2 <- Group2[2:7,]	11

The code setwd(~C:/research/equaltestMI~) on Line 3 sets the working directory as the folder where the data files are stored. The read.table command on Lines 4 and 5 put the sample means and sample covariance matrices of the two groups in Group1.txt and Group2.txt into R environment. The format of the two data files Group1.txt and Group2.txt are provided in **Appendix A**. The argument header is set to be TRUE in order to identify the variable names that are needed to set the model. Lines 6 and 7 then use as.matrix to convert the formats of Group1 and Group2 to matrix as required by lavaan, so that the sample means and covariance matrices extracted from Group1 and Group2 are in the correct formats. Lines 8 to 11 separate the sample means from the sample covariance matrices for each group according to the positions of the values in the data files.

Another argument that is needed by eqMI.main() is the model statement. Since lavaan and semTools are used to compute chi-square and chi-square-difference test statistics, the model syntax is written following the convention of lavaan:

**Table:** 

model <- '	12
AlphabetKnowledge =~ Letter_Name + Letter_Sound	13
PhonologicalAwareness =~ Blending + Elision	14
Spelling =~ Real_Words + Pseudo_Words	15
′	16

where the single quotation marks (can also be double quotation marks) enclose a model statement. The sign =~ is used to indicate the relationship between a latent factor and its manifest indicators/variables. On the left of each =~ is the label for a latent factor and those following =~ are the corresponding manifest variables that loaded onto the latent factor. The manifest variables that load onto the same latent factor are connected by “+.” The names of the latent factors in the model statement cannot duplicate any of the variable names in Group1.txt or Group2.txt.

To perform ET and the projection method for MI with two groups, we supply the following arguments to eqMI.main():

**Table:** 

test <-	eqMI.main(model = model,	17
	sample.nobs = c(78, 174),	18
	sample.mean = list(M1, M2),	19
	sample.cov = list(Cov1, Cov2),	20
	meanstructure = TRUE,	21
	output = 'both',	22
	quiet = FALSE,	23
	equivalence.test = TRUE, adjRMSEA = TRUE,	24
	projection = TRUE, bootstrap = FALSE)	25

The model on Line 17 is the SEM model we defined using the convention of lavaan on Lines 12 to 16. The sample.nobs on Line 18 contains the numbers of observations for the two groups, and more numbers are needed with more groups. The sample.mean on Line 19 is a list of sample means obtained on Lines 8 and 9, and sample.cov is a list of sample covariance matrices. The meanstructure = TRUE on Line 21 is needed if mean structures are involved instead of saturated means. The output = 'both' on Line 22 requires the results of tests of both the mean and covariance structures (steps 1 to 6c in Table 1) be printed out. One can also output the results of only the mean structure or the covariance structure by specifying output = 'mean' or output = 'covariance'. The quiet = FALSE on Line 23 tells the program to print out a summary to R console that contains test statistics and fit measures of all the involved tests as described in Table 1. The arguments equivalence.test = TRUE and adjRMSEA = TRUE on Line 24 tell the program to conduct ET and print out T-size RMSEA and adjusted cutoff values. The arguments projection = TRUE and bootstrap = FALSE on Line 25 tell the program to conduct mean comparison using the projection method. Bootstrap resampling is not invoked in this example due to the absence of raw data. However, bootstrap can be enabled to obtain empirical *p*-values for the tests of equalities of common and specific factors using the projection method once raw data become available, and the details are documented in the online supplementary material.

### 3.3. Output

**Table:** 

---------- Equality of Population Covariance Matrices under NHT ----------				26				
	Chisq	Df	pvalue	27				
fit.pop.cov	48.85008	21	0. 0005261139	28				
29								
---------- Chi-Square and Chi-Square-Difference Test under NHT ----------								30
	Chisq	Df	pvalue	Chisq.diff	Df.diff	pvalue	31	
fit.pop.cov	48.850	21	0.001				32	
fit.configural.g1	4.408	6	0.622				33	
fit.configural.g2	10.641	6	0.100				34	
fit.combine.groups	15.049	12					35	
fit.metric	20.033	15	0.171	4.984	3	0.173	36	
fit.residuals	42.512	21	0.004	22.479	6	0.001	37	
fit.varfactor	54.175	27	0.001	11.663	6	0.070	38	
fit.scalar	23.732	18	0.164	3.699	3	0.296	39	
fit.strong.means	41.066	21	0.006	17.334	3	0.001	40	
fit.strict.residuals	45.968	24	0.004	22.237	6	0.001	41	
fit.strict.means	63.630	27	0.000	17.662	3	0.001	42	
								43
-------------- T-size epsilon, RMSEA, and Adjusted Cutoff Values under ET --------------								44
	epsilon_t	RMESA_t	cut.01	cut.05	cut.08	cut.10	goodness-of-fit	45
fit.pop.cov	0.209	0.141	0.076	0.097	0.121	0.139	poor	46
fit.configural.g1	0.028	0.097	0.116	0.133	0.157	0.175	excellent	47
fit.configural.g2	0.071	0.154	0.116	0.133	0.157	0.175	fair	48
fit.metric	0.049	0.181	0.151	0.164	0.187	0.205	fair	49
fit.residuals	0.140	0.216	0.116	0.133	0.157	0.175	poor	50
fit.varfactor	0.078	0.161	0.116	0.133	0.157	0.175	mediocre	51
fit.scalar	0.040	0.163	0.151	0.164	0.187	0.205	close	52
fit.strong.means	0.125	0.289	0.151	0.164	0.187	0.205	poor	53
fit.strict.residuals	0.138	0.215	0.116	0.133	0.157	0.175	poor	54
fit.strict.means	0.127	0.291	0.151	0.164	0.187	0.205	poor	55
								56
------ Means of Latent and Specific Factors by the Projection Method and under NHT ------								57
	Chisq	Df	pvalue	58				
fit.mvmean	22.388932	6	0.0010292280	59				
fit.common	19.433779	3	0.0002223618	60				
fit.specific	4.015387	3	0.2598074102	61				
Validity Index is 0.9885648				62				
				63				
------ Means of Latent and Specific Factors by the Projection Method and under ET ------								64
	epsilon_t	RMESA_t	cut.01	cut.05	cut.08	cut.10	goodness-of-fit	65
fit.mvmean	0.139	0.215	0.116	0.133	0.157	0.175	poor	66
fit.common	0.137	0.302	0.151	0.164	0.187	0.205	poor	67
fit.specific	0.042	0.168	0.151	0.164	0.187	0.205	fair	68
								69
---------- Cross-group Comparison of Latent Factor Means ----------								70
	latent_1	latent_2	latent_d	SE_d	z_d	71		
AlphabetKnowledge	39.20010	34.77505	−4.42505	1.87963	−2.35422	72		
PhonologicalAwareness	10.50104	8.29014	−2.21090	0.59194	−3. 73503	73		
Spelling	22.14624	17.69643	−4.44981	1.11260	−3. 99946	74		
						75		
---------- Cross-group Comparison of Common Scores ----------						76		
	common_1	common_2	common_d	SE_d	z_d	77		
Letter_Name	39.20010	34.77505	−4.42505	1.87963	−2.35422	78		
Letter_Sound	45.65332	40.49980	−5.15351	2.18906	−2.35422	79		
Blending	10.50104	8.29014	−2.21090	0.59194	−3.73503	80		
Elision	7.11369	5.61597	−1.49772	0.40099	−3.73503	81		
Real_Words	22.14624	17.69643	−4.44981	1.11260	−3.99946	82		
Pseudo_Words	16.45361	13.14762	−3.30600	0.82661	−3.99946	83		
						84		
---------- Cross-group Comparison of Specific Factors ----------						85		
	specific_1	specific_2	specific_d	SE_d	z_d	86		
Letter_Name	6.05990	6.54495	0.48505	0.92562	0.52403	87		
Letter_Sound	−5.20332	−5.61980	−0.41649	0.79478	−0.52403	88		
Blending	0.40896	0.78986	0.38090	0.21495	1.77204	89		
Elision	−0.60369	−1.16597	−0.56228	0.31730	−1.77204	90		
Real_Words	1.73376	1.54357	−0.19019	0.25533	−0.74490	91		
Pseudo_Words	−2.33361	−2.07762	0.25600	0.34367	0.74490	92		

Running the R codes on Lines 17 to 25 generates the above output that has eight parts. Part 1 (Lines 26 to 28) contains the results of testing equality of population covariance matrices under NHT. The package lavaan does not provide such a test so that we developed an R function eqMI.covtest() to perform this test using the method of Lagrange multiplier. Part 2 (Lines 30 to 42) contains the results of MI under the conventional NHT, including the chi-square and chi-square-difference test statistics along with their degrees of freedom and *p*-values. Part 3 of the output (Lines 44 to 55) are the results of MI under ET, consisting of the T-size ϵ_*t*_, RMSEA_*t*_, adjusted cutoff values, and labels of the goodness of fit by comparing RMSEA_*t*_ against the adjusted cutoff values. Note that the results on Line 49 to 55 are based on the chi-square-difference statistics whereas those on Lines 46 to 48 are based on the *T*_*ml*_ statistic as reported in Parts 1 and 2.

Part 4 of the output (Lines 57 to 62) contains the results of testing the cross-group equality of means using the projection method and under NHT. The numbers following fit.mvmean is the results of the Wald test of equality of means of the manifest variables, those following fit.common and fit.specific are the results of the Wald tests of the cross-group equality of means of common and specific factors, respectively. Line 62 contains the value of the validity index according to Equation (12). Part 5 (Lines 64 to 68) contains the results of mean comparison by the projection method and under ET, where the T-size ϵ_*t*_ and RMSEA_*t*_ are based on the Wald statistics reported in Part 4. For each of the tests listed in the output, one can extract details such as parameter estimates and standard errors from the resulting R object test on Line 17.

Parts 6 to 8 (Lines 70 to 92) of the output of eqMI.main() contain parameter estimates, standard errors and the corresponding *z*-scores under the projection approach. Those corresponding to the differences of the estimates across groups are also included. These are the same under NHT and ET.

## 4. Results

It follows from Line 28 that the equality of population covariance matrices is rejected under NHT at level α = 0.05. According to the results on Line 46, we cannot regard the two population covariance matrices as equal under ET unless we can tolerate a model with RMSEA_*t*_ = 0.141. With the adjusted cutoff value for poor model being at 0.139, the model under equal covariance matrices is worse than poor. Consequently, we reject the hypothesis and conclude that the two population covariance matrices cannot be regarded as equal.

We next turn to the components of the measurement models as represented by Figure 1. Under conventional NHT, Lines 33 and 34 indicate that the significance level of the statistic *T*_*ml*_ for group 1 (boys ineligible for FRL) is 0.622, and for group 2 (boys eligible for FRL) is 0.100. One would conclude that configural invariance holds in the population under NHT and move to the next step of the analysis. In contrast, under ET, the goodness of fit for group 1 (Line 47) is excellent with RMSEA_*t*_ = 0.097; but that for group 2 (Line 48) is fair with RMSEA_*t*_ = 0.154. Configural invariance is again established under the condition that we are able to tolerate a model with fair fit or RMSEA_*t*_ = 0.154.

Moving to the next analysis of metric invariance (cross-group equality of factor loading matrices *H*_λ_) under NHT (Line 36), the *p*-value corresponding to the chi-square difference statistic of 4.984 is 0.173, and we conclude that metric invariance holds and move to the next step of the analysis of MI. Under ET, the results on Line 49 indicated that RMSEA_*t*_ = 0.181 and the goodness of fit is fair. Metric invariance is endorsed only we can accept a model of misspecification with RMSEA_*t*_ = 0.181 beyond that in configural invariance.

Following metric invariance, we can next test cross-group equality of variance components (error variances and factor variances-covariances; steps 4a and 5a in Table 1). Alternatively, we can also move to test scalar invariance and cross-group equality of means of latent constructs (steps 4b–6c in Table 1).

Under conventional NHT, with a *p*-value of 0.001 on Line 37, the chi-square-difference statistic suggests that the hypothesis *H*_ψ_ is unlikely to hold. Under ET, results on Line 50 indicate that error variances may not be regarded as equal across the two groups unless we can tolerate a poor model with T-size RMSEA_*t*_ = 0.216.

Move to the mean structure under NHT (Line 39), with a *p*-value of 0.296 for the chi-square-difference statistic, one would conclude that scalar invariance holds in the population. Under ET (Line 52), the T-size RMSEA corresponding to the chi-square-difference statistic for scalar invariance is 0.163, and the model achieved close fit when compared RMSEA_*t*_ against the adjusted cutoff values.

Under NHT, results on Lines 40 to 42 imply that we cannot endorse the cross-group equality of means of the latent constructs (*H*_τ_) nor that of error variances (*H*_ψ_). Thus, strong invariance is achieved but not strict invariance. Results under ET (Lines 53 to 55) also suggest that strict invariance does not hold unless we can tolerate poor models with RMSEA_*t*_ being above 0.20.

Results on Line 59 is the Wald test for cross-group equality of means of the 6 manifest variables. The results for testing the cross-group equality of means of the common and specific factors by the projection method under NHT (Lines 60 and 61) indicate that the two groups have different means of common factors but their means in specific factors might be equal. Consequently, 98.9% of the squared mean differences for manifest variables is due to mean differences in the three latent constructs: alphabet knowledge, phonological awareness, and spelling, indicating that the six variables are good measures of the literacy skills. The results following the projection method under ET (Lines 67 and 68) indicate that we can endorse *H*_*eν*_ and regard the means of the specific factors as being equal across the two groups if a misspecification with RMSEA_*t*_ = 0.168 is tolerable, or be able to accept a fair model. However, we will have to accept a poor model in order to endorse *H*_*eκ*_ or to tolerate a misspecification with RMSEA_*t*_ = 0.302.

Lines 70 to 92 of the output are the results for the means of the latent, common and specific factors, following the projection approach. Those on Lines 70 to 74 indicate that boys eligible for FRL have significantly smaller means of latent traits. As expected, the two groups are significantly different in the mean of each of the six common scores, with those in the low-SES group being uniformly smaller. In contrast, the two SES groups do not show significant differences on any of the six specific factors, implying that most of the cross-group differences in manifest variables are due to those in latent traits.

For this example, the conventional method of NHT endorses both metric invariance and scalar invariance. However, NHT cannot claim that the two properties hold in the population, since it is designed for rejecting the null hypothesis instead of proving that the null hypothesis holds. In contrast, the method of ET did not conclude cross-group equality of either the factor loadings or intercepts. Instead, ET claims that, with probability of 0.95, the difference between the two factor-loading matrices is less than 0.049 as measured by *F*_*ml*_ or less than 0.181 as measured by RMSEA. Similarly, ET claims that, with probability of 0.95, the difference between the two vectors of intercepts is less than 0.040 as measured by *F*_*ml*_ or less than 0.163 as measured by RMSEA. With the projection method, ET claims that with probability 0.95 the two vectors of means of specific factors differ by less than 0.042 as measured by *F*_*gls*_ or less than 0.168 as measured by the corresponding RMSEA. We are able to endorse metric and scalar invariance only if we can tolerate models with fair fit, and the endorsement is attached with a T-size and a probability.

While the statistic *T*_*ml*_ is not significant for the hypothesis of scalar invariance *H*_γ_ in the example, it is rare in practice. The projection method allows us to estimate and compare the means of latent traits as long as metric invariance is endorsed, and a validity index is also provided.

## 5. Conclusion

In this article, we introduced two recently proposed methods, combined the projection-based method and ET, implemented the new methods in an R package, and illustrated the use of the R package via a real data example. We believe that the development will contribute to the use of the cutting-edge methodology in substantive areas where MI is needed in group comparison. In particular, we recommend that researchers report the results of ET together with those under NHT even if they may not want to abandon the method of NHT in studying MI.

We only illustrated ET in the context of MI in this article. ET is equally applicable in other contexts where NHT has been the dominant methodology, especially in areas where models are needed to account for the relationship among the observed variables (e.g., growth curve modeling, time series analysis, item response models) rather than rejecting the null hypotheses. Recent developments for ET in structural equation modeling include Marcoulides and Yuan (2017) and Yuan et al. (2016), where both RMSEA and CFI (Bentler, 1990) can be used for determining the tolerable size of misspecification. ET can also be used for parameter testing, especially when a particular value of the parameter is of special interest (Wellek, 2010).

Throughout the article, we have used RMSEA to quantify the cross-group difference in model parameters. However, Cohen's *d* or standardized mean difference is regularly used in *t*-test and ANOVA. We might adopt Cohen's *d* for ET when quantifying the cross-group differences in the means of latent traits. However, it is not clear how to generalize the standardized mean difference to multiple groups when the covariance matrices of the latent traits are heterogeneous. Correlated latent factors might also cause difficulty with interpretation if we generalize *d* to a multivariate version (Huberty, 2002). Vandenberg and Lance (2000) discussed the pros and cons of different approaches to mean comparison and recommend using overall model fit indices to assess the appropriateness of imposed invariance constraints.

Like any statistical methodology, ET needs a statistic that approximately follows a central/non-central chi-square or another distribution of known form. When such a distribution is not available, especially when conditions are not met (e.g., non-normally distributed data, missing values), alternative statistics other than *T*_*ml*_ might be needed. Bootstrap methodology can also be considered. Further developments are needed in these directions.

## Author contributions

GJ carried out the project, write the example and the initial draft of the article. YM did the program and coding of the software equaltestMI. KY directed the project, and finalized the article in writing.

### Conflict of interest statement

The authors declare that the research was conducted in the absence of any commercial or financial relationships that could be construed as a potential conflict of interest.

## Footnotes

^1^Even if MI does not hold literally, the violation against MI is small enough that we can comfortably ignore it.

^2^The value of the conventional RMSEA is computed according to the value of the observed test statistic *T*_*ml*_ whereas RMSEA_0_ in Equation (5) is related to the value of ϵ_0_ in Equation (3) or ϵ_0*ab*_ in Equation (4), and is used for the purpose of ET.

^3^These cutoff values are necessary for labeling the goodness of model fit but may be of limited scientific value (see Lai and Green, 2016).

^4^Wald statistics are typically formulated via generalized least squares (GLS), and are commonly called GLS statistics in the psychometric literature.

^5^Note that the ncp δ_0_, RMSEA_0_ and RMSEA_*t*_ corresponding to the Wald statistic *T*_*gls*_ might be different from that corresponding to *T*_*ml*_ for a given condition of misspecification. But their difference is tiny unless the model is severely misspecified.

## Appendix A

Data files Group1.txt and Group2.txt used in the real data example

Group1.txt :

**Table:**

	LetterName	LetterSound	Blending	Elision	Real_Words	Pseudo_Words
Mean	45.26000	40.45000	10.91000	6.51000	23.88000	14.12000
LetterName	207.36000	159.09696	32.58864	25.80480	61.77600	45.07488
LetterSound	159.0 9696	280.22760	42.88788	36.74765	76.12348	60.20374
Blending	32.58864	42.88788	18.23290	10.71258	19.05103	14.21910
Elision	25.80480	36.74765	10.71258	20.07040	20.37235	16.70861
Real_Words	61.77600	76.12348	19.05103	20.37235	73.61640	47.42852
Pseudo_Words	45.07488	60.20374	14.21910	16.70861	47.42852	44.35560

Group2 . txt :

**Table:**

	Letter_Name	Letter_Sound	Blending	Elision	Real_Words	Pseudo_Words
Mean	41. 32000	34.8800	9.080000	4.450000	19.24000	11.07000
Letter_Name	295.84000	232.2000	38.995840	20.173880	67.59256	57.77136
Letter_Sound	232.20000	324.0000	43.164000	22.824000	77.95440	60.45840
Blending	38.99584	43.1640	19.009600	9.260204	23.42802	16.27152
Elision	20.17388	22.8240	9.260204	10.048900	15.25404	11.04174
Real_Words	67.59256	77.9544	23.428024	15.254040	64.32040	38.41099
Pseudo_Words	57.77136	60.4584	16.271520	11.041744	38.41099	38.68840
